# Genome-Wide Characterization of *SlABCG* Genes in Tomato Reveals Their Role in Saline–Alkali Tolerance

**DOI:** 10.3390/genes17010019

**Published:** 2025-12-26

**Authors:** Ying Li, Wentao Guo, Hongliang Ji, Weilin Cao, Gaoqing Li, Ruirui Xu, Liming Gan

**Affiliations:** 1 College of Biology and Oceanography, Weifang University, Weifang 261061, China; ying127567@163.com; 2 University of Chinese Academy of Sciences, Beijing 100049, China; g1779367440@163.com; 3 School of Advanced Agricultural Sciences, Weifang University, Weifang 261061, China; wufou888@163.com; 4 The Engineering Research Institute of Agriculture and Forestry, Ludong University, Yantai 264000, China; cwl3237@ldu.edu.cn; 5 Shouguang Vegetable Industry Development Center, Weifang 261061, China; ligaoqing1@163.com; 6 College of Life Sciences, Shandong Agricultural University, Taian 271018, China

**Keywords:** tomato, ABCG, genome-wide, saline-alkali stress

## Abstract

Background: The ATP-binding cassette (ABC) G subfamily, a key member of the ABC protein family, mediates plant stress responses by transporting metabolites across membranes, but its mechanism of action in tomato (*Solanum lycopersicum* L.) remains poorly understood. Methods: We systematically analyzed the evolutionary relationships, structural characteristics, stress-responsive expression patterns, and functional roles in response to saline-alkali stress of the *SlABCG* gene family in tomato, using a combination of approaches including phylogenetic analysis (MEGA), gene structure and motif analysis (GSDS, MEME), cis-acting element prediction, homology analysis, transcriptome analysis, protein-protein interaction prediction, and qRT-PCR validation. Results: We identified a total of 41 *SlABCG* genes from the tomato genome. These genes, together with 43 *ABCG* genes from *Arabidopsis thaliana*, were clustered into five distinct clades. There are 35 collinear gene pairs between the *SlABCG* gene family in tomato and the *ABCG* gene family in *Arabidopsis*, while 39 collinear gene pairs exist among *ABCG* genes within the tomato genome itself.The promoter regions of *SlABCG* genes contain cis-acting elements associated with responses to salicylic acid, low temperature, and gibberellin stresses. Transcriptome sequencing revealed that six *SlABCG* genes responded to saline-alkali stress. Gene regulatory network prediction revealed that multiple genes related to saline-alkali stress were regulated. Expression profile analysis of the 25 upregulated genes revealed that all of them were significantly upregulated during the saline-alkali stress treatment. Conclusions: In summary, our results provide deep insights into the characteristics of the SlABCG subfamily, facilitate the design of effective analysis strategies, and offer data support for exploring the roles of ABCG transporters under different stress conditions.

## 1. Introduction

The ATPase-binding cassette (ABC) proteins are composed of two conserved domains, including the nucleotide-binding domain (NBD) and transmembrane domain (TMD), and their subfamilies are ubiquitous in plants [1,2]. The main function of the membrane-bound transporters encoded by the ABC transporter gene family is to mediate the molecular transport of soluble proteins between different cells or across the plasma membrane [2]. To date, a large number of ABC transporters have been identified in various plants, including *Arabidopsis thaliana*, rice, *Capsicum* spp., soybean, *Linum usitatissimum* L., China rose, grape, and *Camellia sinensis* [3,4,5,6,7,8,9,10,11]. The plant ABC transporter gene family is primarily categorized into eight subfamilies, designated as ABCA to ABCI, with ABCH excluded [12]. Unlike other subfamilies, the ABCG subfamily is arranged according to the structural organization of the nucleotide-binding domain (NBD)–transmembrane domains (TMDs) [13]. Based on the composition of the NBD and TMD, ABCG transporters can be divided into two categories, including the half-size molecular transporter (white–brown complex, WBC) with the NBD-TMD and PDR (pleiotropic drug resistance) arranged in the NBD-TMD-NBD-TMD structure [14,15].

As the largest subfamily of the ABC gene family, the ABCG is involved in various biological processes in plants, such as male reproduction and phytohormone transport [16,17]. During the salt stress treatment of rice, the *ABCG7* mutant exhibits a high degree of leaf wilting, low survival rate, and reduced salt tolerance [18]. AtABCG36 contributes to salt resistance in *Arabidopsis* by a mechanism that includes the reduction of sodium content in plants [19]. Under salt stress conditions, the ABCG proteins in different tissues of *Betula halophila* responded differently. For instance, the expression of *BhABC12* in the xylem and leaves was upregulated; *BhABC14* in the xylem, roots, and leaves showed varying degrees of upregulation; and the expression of *BhABC15* in the xylem and roots was downregulated [20]. The ABCC subfamily member *ZmMRPA6* of maize can enhance its salt tolerance [21]. In transgenic tobacco, the overexpression of *MsABCG1* can enhance plant drought tolerance by regulating the increase of osmolytes and the reduction of lipid peroxidation, and also has the potential to regulate stomata [22]. After double knockdown of the *ABCG17* and *ABCG18* genes, it was found that the transporters encoded by these genes can not only limit stomatal aperture, conductance, and transpiration, but also improve water-use efficiency. Additionally, they can regulate the translocation of abscisic acid (ABA) from the shoot to the root, thereby modulating lateral root development [23]. *VvABCC1* regulates anthocyanin transport, thereby influencing the color of grapes [10]. These studies indicate that members of the ABCG subfamily play a crucial role in regulating plant stress tolerance, growth, and development. Although the *ABCG* gene subfamily in tomato has been identified, its functional mechanisms remain unanalyzed [24].

Abiotic stresses, as major adverse environmental conditions, can reduce plant yield and quality [25]. For tomato, the key factors affecting its yield and quality mainly include extreme temperatures, drought, and soil salinization [26]. Thus, the identification of key genes in tomato that respond to abiotic stresses not only facilitates the elucidation of its tolerance mechanisms but also provides a crucial theoretical basis and practical guidance for the breeding of stress-resistant plants, thereby holding significant importance. This study analyzed the characteristics of the ABCG subfamily in tomato, including their phylogenetic relationships, gene structures, cis-acting elements, and interaction networks. By detecting the expression of the *ABCG* gene under different abiotic stresses, we identified candidate genes with the potential to tolerate various abiotic stresses. This study contributes to revealing the potential functions of tomato *ABCG* genes under abiotic stresses and ultimately provides a theoretical basis and practical reference for the cultivation of tomato with broad-spectrum resistance.

## 2. Materials and Methods

### 2.1. Plant Materials and Treatments

This study used seeds of the Micro-Tom tomato, which were first germinated under conditions of 25~28 °C, approximately 75% relative humidity, 12/12 h (light/dark) photoperiod achieved with natural light and augmented with supplemental lights (400 W high-pressure sodium lamp) and 1400 μmol·s^−1^·m^−2^ average photosynthetic photon flux density across replications for daytime hours. After the seedlings developed 4~6 true leaves, those with uniform growth were transplanted into plastic containers containing new Hoagland nutrient solutions (pH 5.5~6.5), with their roots subjected to dark treatment. At this point, the tomato seedlings were randomly divided into two groups: the control group (CK) was cultured only with Hoagland nutrient solution; the treatment group (N) was supplemented with 300 mM mixed saline–alkali solution in Hoagland nutrient solution (the molar ratio of each component was NaCl:Na_2_SO_4_:NaHCO_3_:Na_2_CO_3_ = 1:9:9:1, pH = 8.6 ± 0.1). The solution was replaced every 2 d. Samples were collected at 0 h, 4 h, and 8 h after treatment, with 3 biological replicates set for each treatment.

### 2.2. RNA Extraction and RNA-seq

Total RNA was extracted from tomato seedlings under saline–alkali stress using the Trizol reagent (TaKaRa, Shiga, Japan), following the manufacturer’s instructions. The Agilent 2100 Bioanalyzer (Agilent Technologies, Santa Clara, CA, USA) was used to analyze the integrity, quality, and concentration of the extracted RNAs. High-quality RNA samples were selected to construct cDNA libraries using the VAHTS Universal V10 RNA-seq Library Prep Kit (Vazyme, Nanjing, China), and sequencing was performed on the Illumina HiSeq™ 2500 platform (Shanghai, China) based on the standard protocols.

### 2.3. Transcriptome Data Collection

In this study, tomato transcriptome data under different stress treatments were retrieved from the Sequence Read Archive (SRA) of the National Center for Biotechnology Information (NCBI). The BioProject numbers of the tomato transcriptome data under different treatments are as follows: ABA treatment is PRJNA947059 (the leaves of the tomato plants that had grown four true leaves were collected after being sprayed with 10 mL of 100 μM ABA). Light treatment is PRJNA270083—tomato plants underwent one of four treatments: control (mock, nightly dark environment with MgCl_2_ treatment), RL (nightly red light treatment with MgCl_2_ treatment), DC3000 (nightly dark environment with DC3000 inoculation), and RL + DC3000 (nightly red light treatment with DC3000 inoculation); leaves were collected from the plants at 5:00 AM the next day after treatment. Low-temperature treatment is PRJNA484882 (tomato leaves were treated at 4 °C) and SA treatment is PRJNA846581 (after 3 weeks of growth, when the tomato plants had 5 to 6 leaves, they were sprayed with 0.5 mM SA and the leaves were collected).

### 2.4. Transcriptome Data and Differential Expression Analysis

The collected and sequenced transcriptome data were analyzed in this study. Firstly, the Trimmomatic software (v0.39) was used to remove low-quality bases and adaptors [27]. All clean reads of the samples were aligned to the tomato reference genome (SL3.0) using HISAT (v2.1.0) [28]. The StringTie software (v2.1.3) was employed to assemble transcripts in each sample, which were then aligned to the tomato reference genome using Gffcompare (v0.11.2). The Salmon software (v0.14.1) was utilized to quantify all transcripts, with expression levels represented by TPM [29]. For differential expression analysis, the R packages DESeq2 (v1.30.0) and edgeR were used for data with and without biological replicates, respectively [30,31]. The identification criteria were set as false discovery rate (FDR) < 0.05 and log_2_ (fold change) > 1.

### 2.5. Phylogenetic, Gene Structure, and cis-Regulatory Element Analysis

An unrooted phylogenetic tree of ABCG protein sequences from tomato and *Arabidopsis* was constructed using the MEGA 7.0 software with the neighbor-joining method, and the bootstrap value was set to 1000 [32]. We modified the phylogenetic tree using Evolview [33]. In this study, genomic sequences and the corresponding coding nucleotide sequences were downloaded from the tomato genome database, and the intron–exon distribution of *ABCG* genes was analyzed using the Gene Structure Display Server (GSDS) [34]. Conserved motifs were predicted with the MEME server [35], and cis-regulatory elements in the promoter regions of the *ABCG* genes were predicted via the PlantCARE database [36]. In this study, the 2 kb sequence upstream of the *ABCG* genes on the chromosome was defined as the promoter region.

### 2.6. Chromosomal Distribution and Gene Duplication Analysis

The distribution of *ABCG* genes on tomato chromosomes was visualized using the TBtools software. To detect gene duplication events and collinear relationships, the TBtools software was also employed to analyze *ABCG* transporter genes in tomato, potato, and *Arabidopsis* [37].

### 2.7. Functional Enrichment and Prediction of Tomato ABCG Protein Interaction Network

The KOBAS server was used to perform a KEGG (Kyoto Encyclopedia of Genes and Genomes) enrichment analysis [38]. The online software STRING 12.0: https://cn.string-db.org/ (accessed on 1 April 2025) was used to predict the ABCG protein interaction network. The interaction network was visualized using the Cytoscape software (V3.10.3) [39].

### 2.8. Quantitative Real-Time PCR Validation

The synthesized cDNA templates were used to perform qRT-PCR with 25 gene-specific primers (Table S8) on a ViiA7 system (ABI) using the SYBR Green I (TB Green Premix Ex Taq II, TaKaRa). The cycling conditions were as follows: 95 °C for 30 s, 40 cycles of 95 °C for 10 s, and 60 °C for 30 s. Melt curves confirmed specificity. The 2^−ΔΔCt^ method (*n* = 3 biological replicates) was used to calculate relative expression levels, and GAPDH was used as the reference gene. Error bars represented the standard error of the mean (SEM). Statistical differences were analyzed by the one-way ANOVA, which was used to analyze statistical differences on the GraphPad Prism 8.0 (* *p* < 0.05, ** *p* < 0.01, *** *p* < 0.001).

## 3. Results

### 3.1. Characterization of SlABCG Genes

**Figure 1:**
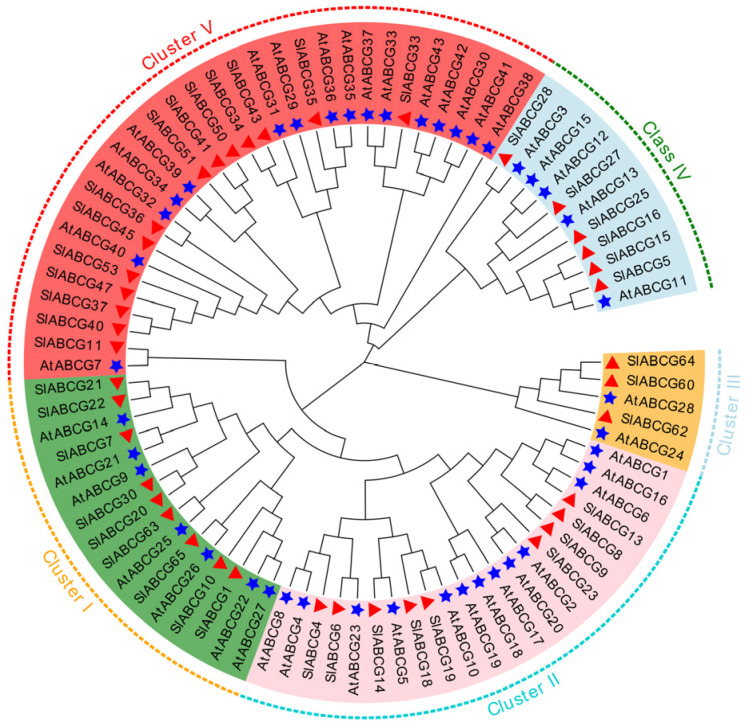
Phylogenetic reconstruction of ABCG proteins. The evolutionary tree was obtained using the neighbor-joining method (MEGA7) with ABCG protein sequences from tomato and *Arabidopsis*. Bootstrap support values (1000 replicates) are displayed at branch nodes. Five-pointed stars represent *Arabidopsis* genes; triangles represent tomato genes.

Based on previous research, we identified 41 genes belonging to the ABCG family from the tomato reference genome Solanum_lycopersicum.SL3.0 (Table S1). The lengths of proteins encoded by the tomato *ABCG* genes exhibited significant variation, ranging from 597 amino acids (SlABCG4) to 4249 amino acids (SlABCG50). Further analysis revealed that the molecular weights of these proteins were in the range of 66.46 kDa to 482.64 kDa, with isoelectric points distributed between 5.98 and 9.28 (Table S1). To clarify the evolutionary relationships among members of the tomato ABCG family, a phylogenetic tree was constructed in this study using full-length protein sequences of 41 tomato *ABCG* genes and 43 *Arabidopsis ABCG* genes. Phylogenetic analysis was performed via the neighbor-joining method with 1000 bootstrap replicates to assess branch reliability, and the results revealed that these ABCG family proteins could be divided into five distinct clusters (Figure 1). In all clusters, members of the tomato ABCG family showed conserved orthology, such as SlABCG8 and SlABCG9 in cluster II and SlABCG15 and SlABCG16 in cluster IV. In addition, cluster V exhibited the highest diversity, containing 16 *Arabidopsis ABCG* genes and 14 tomato *ABCG* genes (Figure 1). This phylogenetic topology indicated that the ABCG family has both lineage-specific diversification events and conserved evolutionary patterns.

### 3.2. Analysis of Gene and Protein Structures of SlABCGs

**Figure 2:**
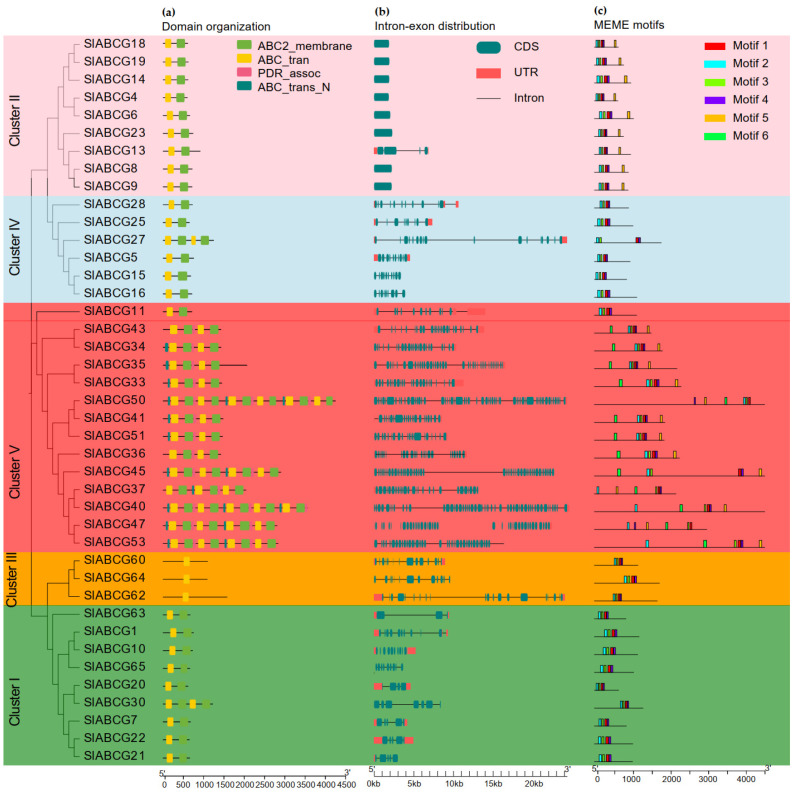
Gene and protein structure analysis of the tomato ABCG transporter subfamily. (**a**) Identification of the conserved domain of SlABCG proteins. (**b**) Gene structure of the *SlABCG* genes, 5′ to 3′ direction indicates the orientation of the nucleotide sequence, and, at the bottom, the scale is the length of the nucleotides (bp). (**c**) Conserved motifs of the SlABCG proteins.

An analysis of the conserved domains in ABCG transporters demonstrated that four conserved domains were present in the two complexes, among which, the first complex harbors either nucleotide-binding domains (NBDs), transmembrane domains (TMDs), or a combination of both (Figure 2a). These genes contain 1 to 58 exons and were distributed across multiple distinct chromosomes. The intron–exon distribution pattern indicates that closely related *ABCG* genes typically shared similar gene structures (Figure 2b). A motif analysis identified a total of six conserved motifs, and their distribution patterns were highly diverse, which verified their phylogenetic classification (Figure 2c). A MEME analysis also revealed that among the six conserved motifs, four overlap with the domains of ABC trans (NBD) and ABC membrane (TMD). Motif 2 and motif 3 belong to the ABC_trans domain, while motif 5 and motif 6 belong to the ABC2_membrane domain (Figure S1).

### 3.3. Synteny and Colinearity Analysis of SlABCG Genes

**Figure 3:**
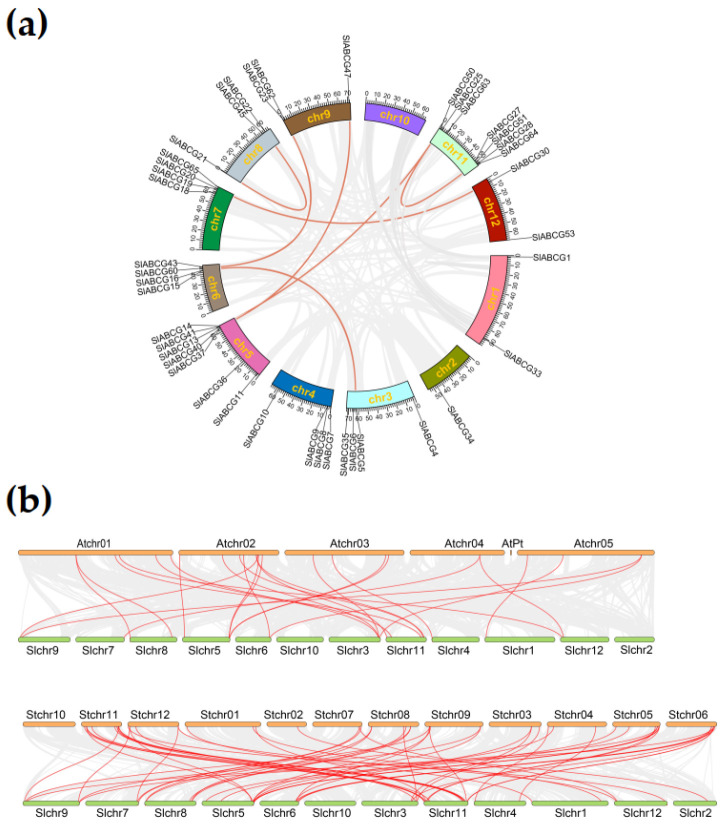
Gene duplication analysis of the SlABCG subfamily. (**a**) Synteny analysis of the tomato ABCG subfamily on different chromosomes. Different color lines represent segmental duplication pairs of SlABCG between chromosomes. (**b**) Synteny relationship analysis of SlABCG between tomato, potato, and *Arabidopsis*. The red lines represent collinear gene pairs and gray lines represent the collinear blocks.

To further investigate the relationships among tomato *ABCG* genes, we performed a genome-wide collinearity analysis of duplication events within this family. The results revealed that the 41 *SlABCG* genes were unevenly distributed across 11 chromosomes, with the highest number of genes being found on chromosomes 5 and 11 (7 genes each). A total of seven segmental duplication pairs were identified in the tomato ABCG subfamily, randomly distributed across eight chromosomes (Figure 3a). This pattern suggested that segmental duplication events may have played a dominant role in driving *ABCG* gene expansion in the tomato genome. Furthermore, to further understand the duplication events of *SlABCG* genes, a collinearity map of the *ABCG* homologous genes among tomato, *Arabidopsis*, and potato was constructed. The results showed that 21 *SlABCG* genes formed 35 collinear gene pairs with 24 *AtABCG* genes, and 21 *SlABCG* genes formed 39 collinear gene pairs with 27 *SlABCG* genes (Figure 3b and Table S2). This finding contributed to exploring the evolutionary relationships among species and predicting gene functions.

### 3.4. Expression Patterns of SlABCGs Under Abiotic Stress

**Figure 4:**
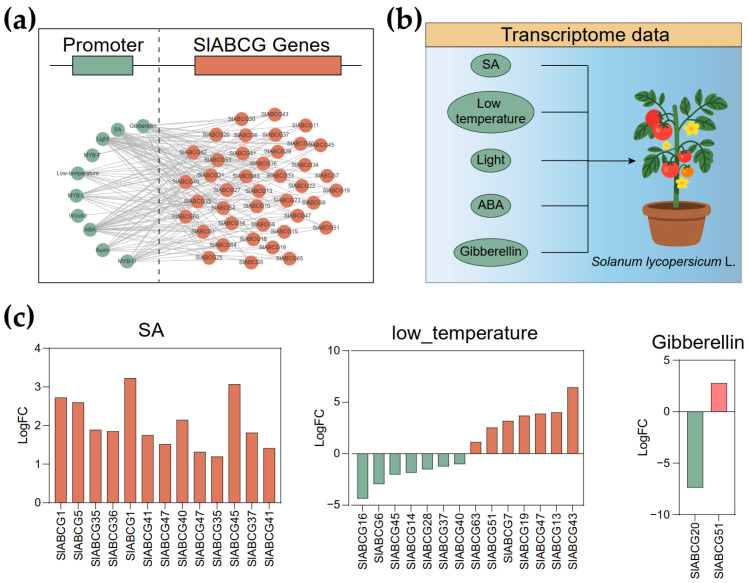
Expression patterns of *SlABCGs* under abiotic stress. (**a**) *Cis*-element prediction for the *SlABCG* gene. (**b**) The transcriptome data of tomato under different stresses, including salicylic acid (SA), low temperatures, light, gibberellin, and abscisic acid (ABA). (**c**) Differentially expressed *SlABCG* genes of tomato under the stresses of SA, low temperatures, and gibberellin.

To further elucidate the regulatory mechanism of *ABCG* genes, we analyzed the promoter sequences of all these genes using the PlantCARE software. A series of cis-elements associated with multiple abiotic stresses were identified, including those responsive to ABA, wound, auxin, gibberellin, light, low temperatures, and SA (Figure 4a and Figure S2). To further validate whether these abiotic stresses induce the expression of *SlABCG* genes, we obtained transcriptomic datasets of tomato plants treated with ABA, gibberellin, light, low temperatures, and SA from the NCBI database (Figure 4b). Subsequently, we conducted a comprehensive analysis of their expression patterns (Table S3). Differential expression analysis revealed that thirteen, seven, and one *SlABCG* genes were upregulated in response to SA, low-temperature, and gibberellin treatments, respectively (Figure 4c). In contrast, no *SlABCG* genes showed significant changes under light or ABA treatments. Intriguingly, seven and one *SlABCG* genes were downregulated during low-temperature and gibberellin treatments, respectively. These results suggest that *SlABCG* genes may play roles in tomato responses to low temperatures and SA.

### 3.5. Function of SlABCG in Tomato Under Saline–Alkali Stress

**Figure 5:**
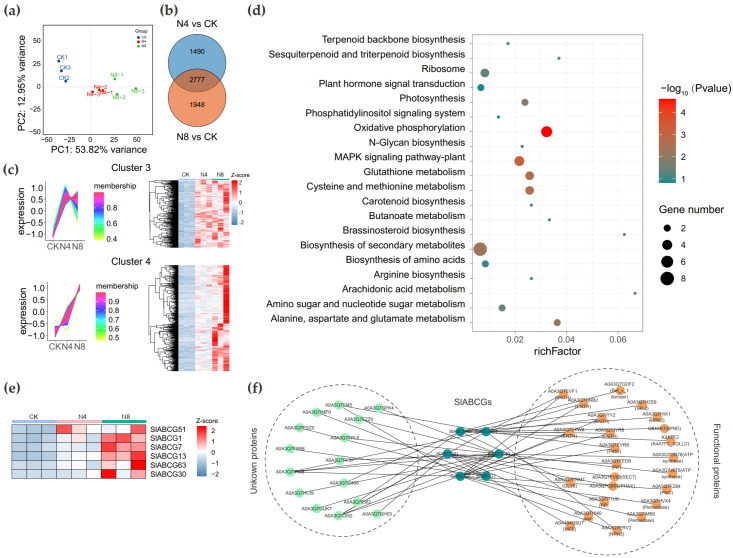
Expression pattern analysis of *SlABCG* genes in tomato under saline–alkali stress. (**a**) Principal Component Analysis (PCA) of all samples. (**b**) The distribution of differential expression genes between N8 vs. CK and N4 vs. CK. (**c**) Expression trend analysis was performed using the R package TCseq and the differentially expressed genes (DEGs) with sustained upregulation are displayed. (**d**) KEGG enrichment analysis of DEGs with sustained upregulation. The size of the circles represents the gene number and from green to red represents −log_10_ (*p* value). (**e**) The heatmap represents the expression pattern of differential expression in *SlABCG* genes with sustained upregulation. (**f**) The String server was used to predict the interaction network of *SlABCG* genes. These interactions occur at the cell membrane.

Studies have demonstrated that the ABCG family plays a crucial role in plant responses to salt stress. We performed transcriptome sequencing on saline–alkali stress-treated tomato plants. Specifically, we employed RNA-Seq technology to sequence the transcriptomes of tomato seedlings subjected to saline–alkali treatment for 0 h (control, CK), 4 h, and 8 h. This yielded 2.03 Gb of clean reads, with the percentage of Q30 bases exceeding 97.0% and the GC content being approximately 42.35% (Table S4). The correlation analysis revealed a high degree of consistency between replicate data at the same treatment time points (Figure 5a). After excluding genes with Transcripts Per Kilobase Million (TPM) < 1 in all samples, a total of 21,165 genes were retained (Table S5). Differentially expressed genes (DEGs) were identified with the criteria of FDR < 0.05 and |log2FC| ≥ 1. Compared with the control (CK), 1561; 2862 upregulated DEGs and 1216; 1863 downregulated DEGs were identified in the 4 h and 8 h groups, respectively (Table S6). A total of 2777 genes were differentially expressed in both the 4 h and 8 h groups (Figure 5b). After analyzing the expression trends of co-DEGs, four distinct expression patterns were identified (Figure S3). Among these, genes in cluster 3 and cluster 4 exhibited increased expression levels at 4 h and 8 h compared to those in the CK group (Figure 5c). The KEGG enrichment analysis revealed that genes in these two clusters were enriched in multiple pathways, including oxidative phosphorylation, the plant-specific MAPK signaling pathway, and glutathione metabolism (Figure 5d, Table S7). The two clusters contain a total of six *SlABCG* genes, with cluster 3 harboring one (*SlABCG51*) and cluster 4 containing five genes (*SlABCG1*, *SlABCG7*, *SlABCG13*, *SlABCG63*, and *SlABCG30*) (Figure 5e). To further characterize the functions of these six *ABCG* genes, we utilized the STRING database to predict their protein interaction networks. Results revealed that these ABCG proteins interact with multiple proteins. In particular, SlABCG7 exhibited interactions with 13 proteins, including peroxidases, ENTH domain-containing proteins, and cytochrome P450 enzymes (Figure 5f).

### 3.6. SlABCG Genes Regulating the Saline–Alkali Stress Response in Tomato

**Figure 6:**
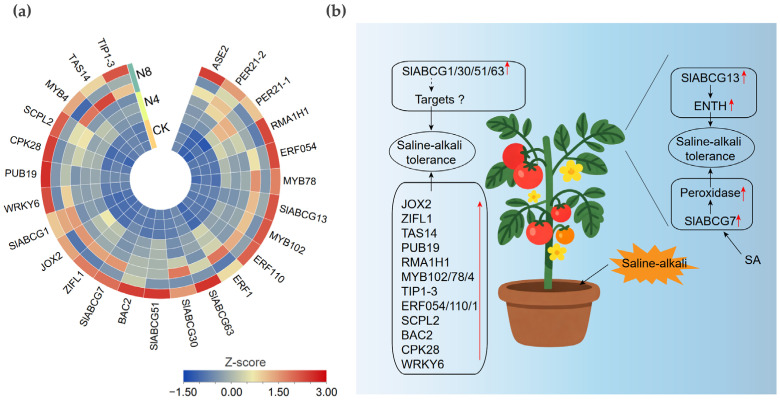
Verification of expression patterns and establishment of a saline–alkali tolerance model in tomato. (**a**) Heatmap of the relative expression levels of the upregulated genes in tomato at different times after saline–alkali treatment. Each column represents a sample, and each row represents a gene. Colors indicate standardized gene expression values (Z-score values), with blue and red denoting low and high transcript abundances, respectively. (**b**) A working model for tomato-responding saline–alkali tolerance. Red arrows indicate the upregulation of gene expression or protein activity.

To further verify the gene response mechanisms to saline–alkali stress, we selected the upregulated *SlABCG* family genes, partial target genes, and other related genes for expression pattern analysis. Quantitative real-time PCR (qRT-PCR) results showed that all genes were upregulated during the saline–alkali stress treatment (Figure 6a and Figure S4). Transcriptome sequencing data revealed that four genes, namely *SlABCG1*, *SlABCG30*, *SlABCG51*, and *SlABCG63*, were significantly upregulated, while their predicted target genes showed no response to saline–alkali stress. This suggested that these *SlABCG* genes may enhance the saline–alkali tolerance of tomato by regulating other target genes. In addition, both *SlABCG13* and its target gene *ENTH* exhibited upregulated expression patterns under saline–alkali stress, indicating that SlABCG13 might improve the saline–alkali tolerance of tomato by promoting the expression of *ENTH*. Notably, the *SlABCG7* gene not only significantly increased the expression levels of two peroxidase genes but its own expression was also induced by salicylic acid (SA). This implies that SlABCG7 may be involved in the SA-mediated mechanism underlying the enhanced saline–alkali tolerance of tomato (Figure 6b).

## 4. Discussion

ABC transporters are a class of widely existing membrane-bound proteins, present in plants, animals, and prokaryotes [4,40,41,42]. From a phylogenetic perspective, the ABC gene family can be divided into different subfamilies, including the ABCA~ABCI subfamilies but excluding the ABCH subfamily [24,43,44]. Among them, the ABCG protein subfamily has a large number of members and plays a key role in multiple signaling pathways involved in plant responses to abiotic stress [18,22,44]. Previous studies have demonstrated that ABCG represents the largest subfamily within the plant ABC gene family, while its functional characterization remains to be addressed [2].

In this study, we identified the ABCG subfamily genes in tomato and conducted an in-depth analysis of their functional mechanisms in response to corresponding abiotic stresses in tomato. Previous studies identified 70 *ABCG* genes in the tomato reference genome [24]. However, using the IDs of these 70 genes, the present study retrieved only 41 *ABCG* genes in the new version of the tomato reference genome. This discrepancy may be attributed to the removal of false positive genes, non-functional pseudogenes, and redundantly annotated genes in the updated genome assembly. A phylogenetic analysis of the ABCG subfamily across plant lineages has revealed several intriguing insights [45]. Our evolutionary analysis classified tomato *ABCG* genes into five clusters based on the evolutionary relationships of *Arabidopsis* ABCG proteins. We hypothesize that proteins with a common ancestral origin may share similar functions. This suggests that SlABCGs clustered with *Arabidopsis* homologs may have certain functional similarities, which requires further verification.

Gene duplication within a single chromosome, between chromosomes, or even across the entire genome may be a major driver for the formation of genetic diversity during genome evolution [46]. Our results showed that in the tomato genome, segmental duplication events of the ABCG subfamily were relatively frequent. Furthermore, we also compared the gene duplication events of ABCG subfamily genes in the tomato genome with those in the *Arabidopsis* and potato genomes. A total of 35 segmental duplication pairs were identified between the tomato and *Arabidopsis* genomes, involving 21 *SlABCG* genes and 24 *Arabidopsis ABCG* genes. When comparing the tomato and potato genomes, 39 segmental duplication pairs were found, containing 21 *SlABCG* genes and 27 potato *ABCG* genes. The majority of the members in the ABCG transporter subfamily are generated through segmental gene duplication, which suggests that segmental duplication might be the main driving force underlying the evolution of ABCG transporters in the tomato genome.

In the process of plant response to abiotic stresses, members of the ABCG subfamily are involved in the regulation of numerous biological processes. Overexpression of the *ABCG1* gene from *Medicago sativa* (*MsABCG1*) in tobacco can significantly enhance the drought tolerance of the plants, as well as increase stomatal density and reduce stomatal diameter [22]. In *Cajanus cajan*, low temperatures can induce the expression of the *CcABCG28* gene, while drought and aluminum stress can induce the upregulated expression of the *CcABCG7* gene [47]. The *ABCG* gene *LkABCG40* from *Larix kaempferi* may enhance the resistance of transgenic tobacco by inhibiting the expression of *WRKY* genes [47]. Identifying transcriptional regulatory elements in the promoter region is a primary strategy for predicting gene function and regulation [48].

In this study, we identified multiple cis-regulatory elements that bind to the *SlABCG* gene promoter, such as light- and low-temperature-responsive elements, as well as ABA-, SA-, and gibberellin-responsive elements. We obtained transcriptome data of tomatoes treated with light, low temperature, ABA, SA, and gibberellin, respectively, and reanalyzed these data. Differential expression analysis revealed that SA, low temperatures, and gibberellin can induce an increase in the transcriptional level of the *SlABCG* gene. SA plays a key role in plant disease resistance, but research on abiotic stress is relatively scarce [49,50]. For instance, high air humidity can inhibit the SA signaling pathway and the expression of *NPR1*, thereby suppressing plant defense capacity [51]. Rice SA hydroxylase genes (*OsSAHs*) exhibit SA-catalyzing activity in vitro. The knockout of *OsSAH2* and *OsSAH3* enhances plant resistance to hemi-biotrophic and necrotrophic pathogens, whereas the overexpression of each *OsSAH* gene increases plant susceptibility to these pathogens [52]. These reports indicated that the *ABCG* gene identified in this study—whose promoter contained SA-binding sites and which was differentially upregulated—may participate in the disease defense mechanism of tomato through the SA signaling pathway. We also found that low temperatures can induce increased transcriptional levels of seven *ABCG* genes. To date, there have been only a few studies on the mechanism of ABCG in plant tolerance to low temperatures. For instance, in *Cajanus*, low temperatures can induce the upregulated expression of *CcABCG* [46]. This suggested that these low temperature-induced *ABCG* genes, which have low-temperature binding sites in their promoters, were highly likely to be involved in plant responses to low-temperature stress, and their molecular mechanisms requires further verification. Furthermore, we found that gibberellin can induce the upregulated expression of one *ABCG* gene; however, the promoter region of this *ABCG* gene lacked gibberellin-responsive elements. Thus, the regulatory relationship between gibberellin and the *SlABCG* gene remains to be further explored.

Numerous studies have shown that *ABCG* genes play a crucial role in plant responses to salt stress. Therefore, we performed transcriptome sequencing on tomato plants subjected to saline–alkali stress. Compared with the CK group, there were 2777 common differentially expressed genes at 4 h and 8 h. Among these genes, 1563 DEGs showed a continuous increase in transcription levels during saline–alkali stress treatment. The KEGG enrichment analysis revealed that these genes are involved in multiple pathways, such as oxidative phosphorylation, MAPK signaling pathway–plant, and photosynthesis, indicating that these pathways are involved in plant responses to saline–alkali stress. There were six *SlABCG* genes in these continuously upregulated genes. A further interaction network analysis showed that these SlABCG proteins interact with various functional proteins, such as SlABCG7 with two genes encoding peroxidase, and SlABCG63 with MPK2. Studies have demonstrated that the *WRKY107* gene in maize can regulate the expression of peroxidase (ZmPOD52) by directly binding to the promoter of maize *ZmPOD52*, thereby regulating the salt tolerance of maize [53]. In addition, MAPK cascades also play a key role in enhancing plant salt tolerance [54]. Under salt stress conditions, exogenous application of the MAPK phosphorylation inhibitor SB203580 reduces the levels of endogenous jasmonic acid (JA), ABA, and ethylene, while decreasing O_2_^−^ and hydrogen peroxide (H_2_O_2_), and lowering the activity of antioxidant enzymes [55]. These findings suggested that SlABCG may form an interaction network with the aforementioned functional proteins to jointly regulate plant tolerance to salt stress. This study provides valuable insights for in-depth investigations of the response mechanisms of the tomato ABCG family to abiotic stress, which is of great significance for improving tomato tolerance and yield.

The present DEG analysis provides only an averaged view of transcriptional changes, which may obscure cell-type-specific and spatially resolved responses. Plant organ-level outcomes emerge from integrated cell-to-cell communication, and the effect of a treatment strongly depends on its position within the hormetic window. Therefore, while our results offer a useful overview, future studies should carefully define treatment conditions and employ more precise in situ approaches to capture the complex spatiotemporal dynamics of hormetic responses.

## 5. Conclusions

In this study, we systematically characterized the *ABCG* gene family using bioinformatics tools, encompassing analyses of evolutionary relationships, gene structures and protein-conserved motifs, chromosomal distribution, the prediction of cis-acting elements, and expression changes in each gene under different treatments. The 41 identified *ABCG* transporter genes could be divided into five clusters, and variations in their gene structural characteristics supported the evolutionary relationships revealed by phylogeny. The prediction of cis-acting elements indicated that *SlABCG* genes might be regulated by various stresses, which was further confirmed by transcriptome data showing that *SlABCGs* were regulated by gibberellin, low temperatures, and SA. The transcriptome sequencing analysis of tomato saline–alkali stress revealed that six *SlABCG* genes had the potential to regulate plant responses to saline–alkali stress, and the interaction network prediction demonstrated that these genes regulated multiple saline–alkali stress-related genes such as peroxidase and MPK2. This information was crucial for enhancing tomato breeding for resilience to short-term hydroponic failures under accidental saline–alkali stress.

## Data Availability

The raw data supporting the conclusions of this article will be made available by the authors on request.

## Supplementary Materials

The following supporting information can be downloaded at https://www.mdpi.com/article/10.3390/genes17010019/s1. Figure S1: Conserved protein sequences of 6 motifs identified in the SlABCG subfamily. Motif scans and sequence logos were generated in the MEME server. Figure S2: *Cis*-element prediction of the *SlABCG* gene. Figure S3: Expression trend analysis was performed using the R package TCseq. Figure S4: Gene expression of 6 *SlABCG* genes, 2 targets, and 17 other key genes in tomato at different times after saline–alkali treatment. Table S1: Characteristics of *SlABCG* genes and the encoded proteins. Table S2: *ABCG* homologous genes between tomato and *Arabidopsis* or potato. Table S3: The expression patterns of tomato genes under abiotic stress. Table S4: RNA-seq analysis of tomato under saline–alkali stress. Table S5: Expression levels of tomato genes. Table S6: Differentially expressed analysis of tomato under saline–alkali stress. Table S7: KEGG pathway enrichment analysis of common DEGs between N4 vs. CK and N8 vs. CK. Table S8: The gene-specific primers.
