# Supplementary figures and images for: Genome-Wide Characterization of SlABCG Genes in Tomato Reveals Their Role in Saline–Alkali Tolerance
Source: Genes (Basel). 2025 Dec 26;17(1):19. doi: 10.3390/genes17010019 (PMC12841099; doi:10.3390/genes17010019)

Motif 1

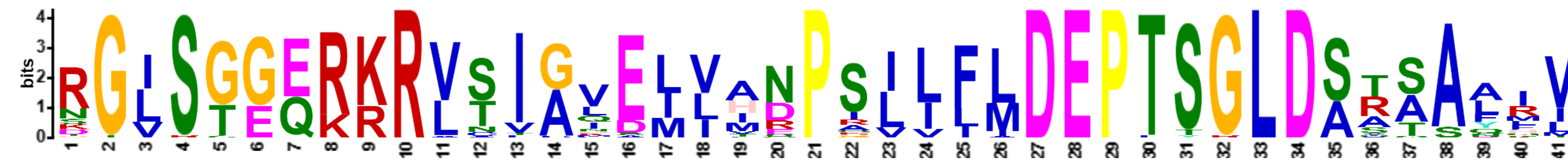

Motif 2

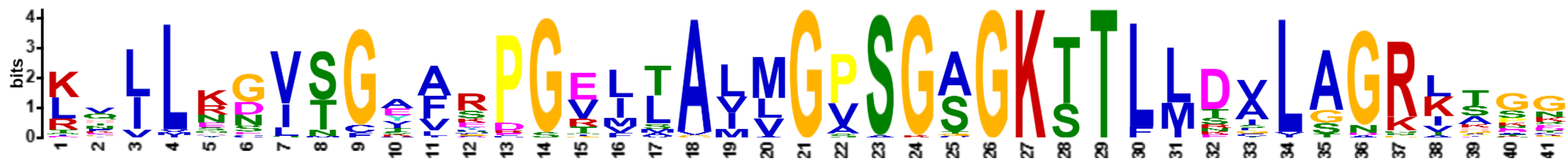

Motif 3

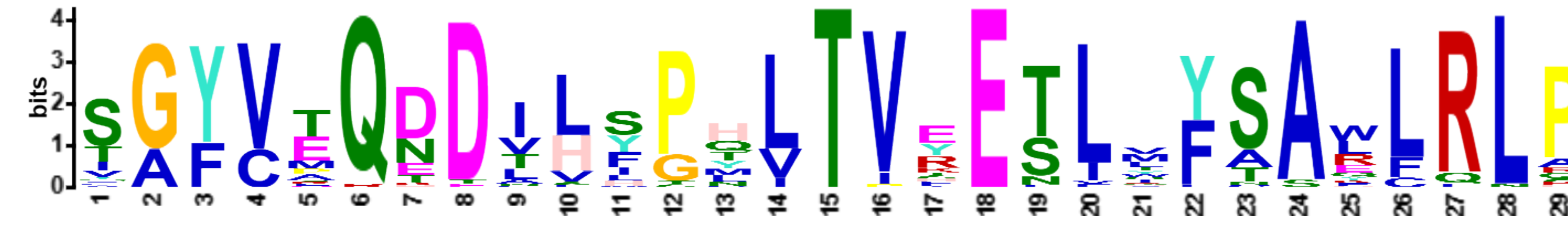

Motif 4

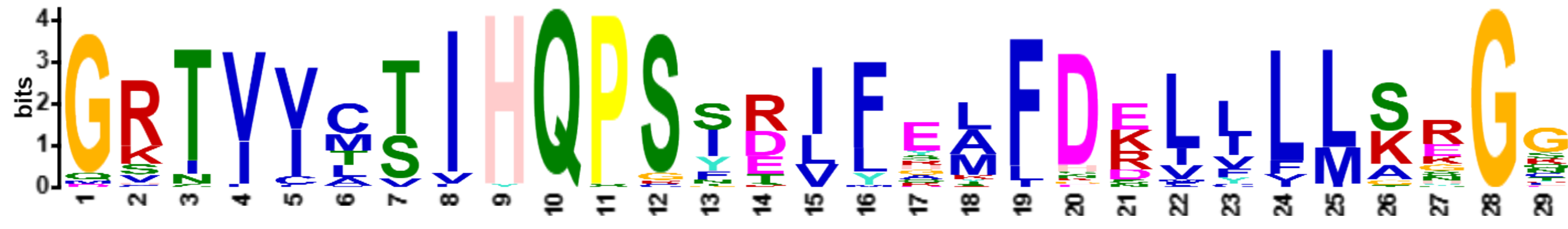

Motif 5

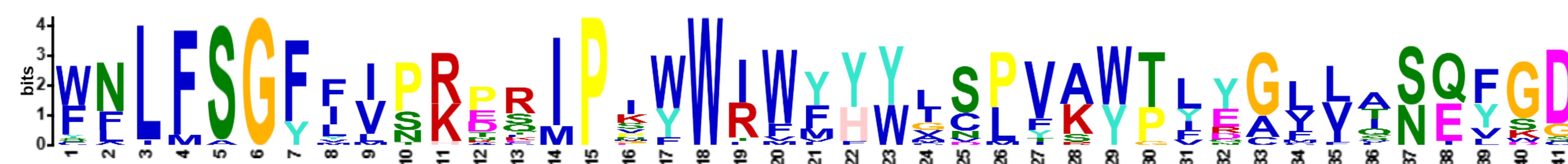

Motif 6

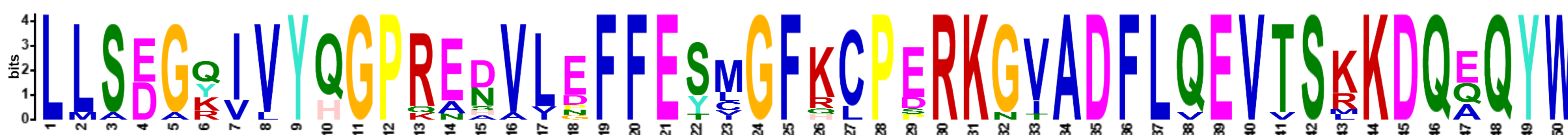

Supplement: Supplementary file 1 [file genes-17-00019-s001.zip › genes-4063062-supplementary/Supplementary File/Figure S1.pdf]

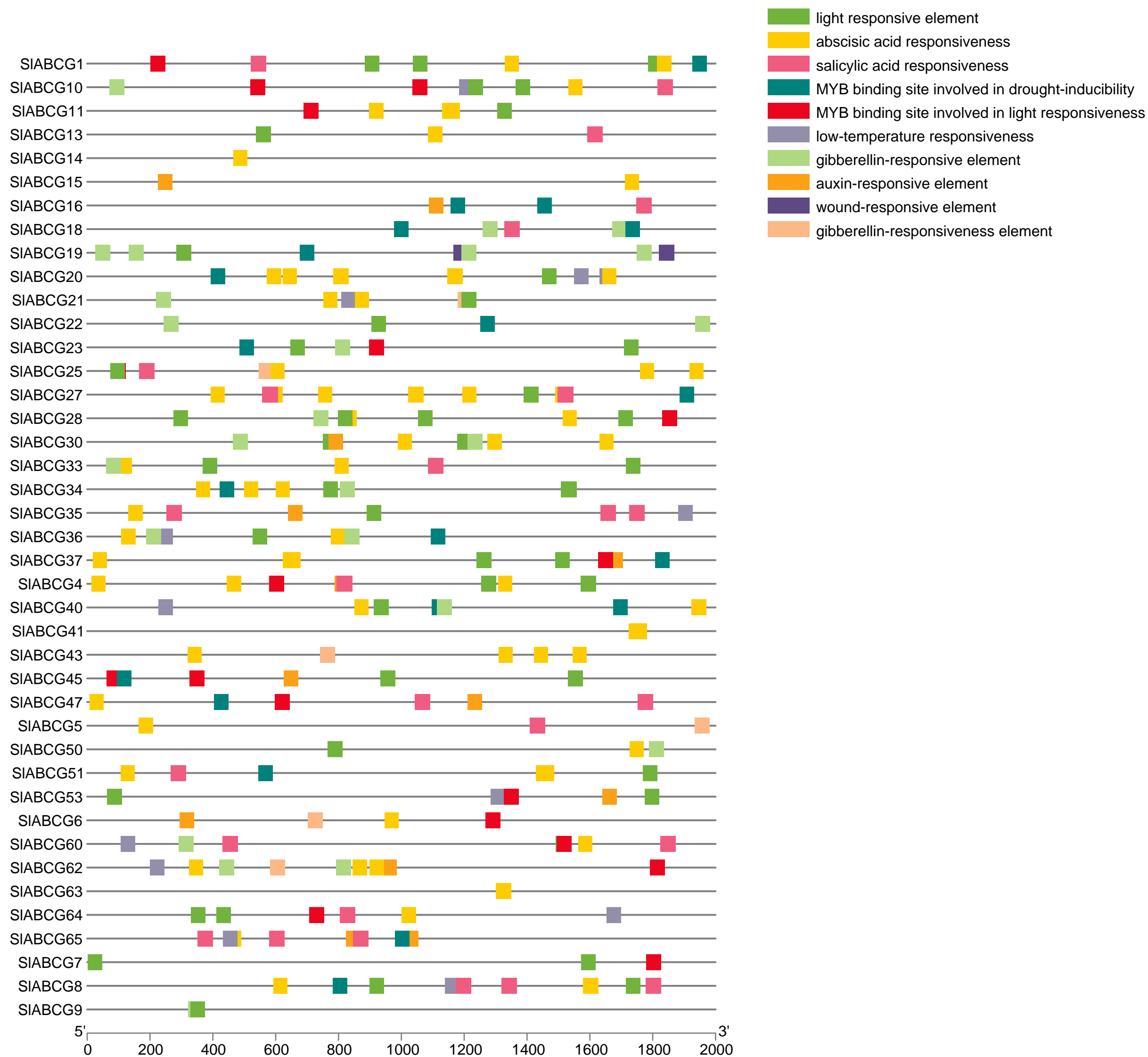

Supplement: Supplementary file 1 [file genes-17-00019-s001.zip › genes-4063062-supplementary/Supplementary File/Figure S2.pdf]

### Cluster 1

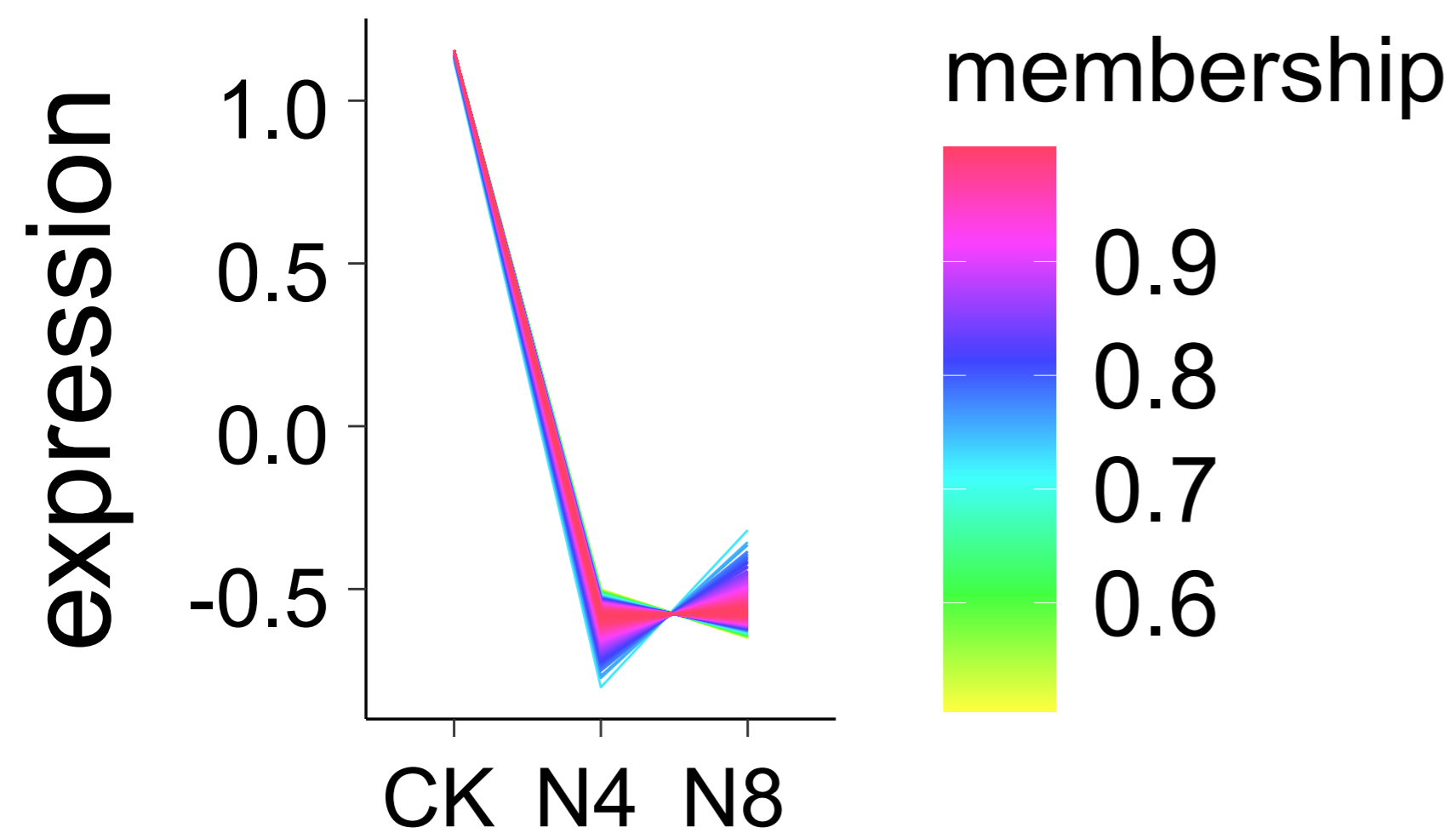

### Cluster 3

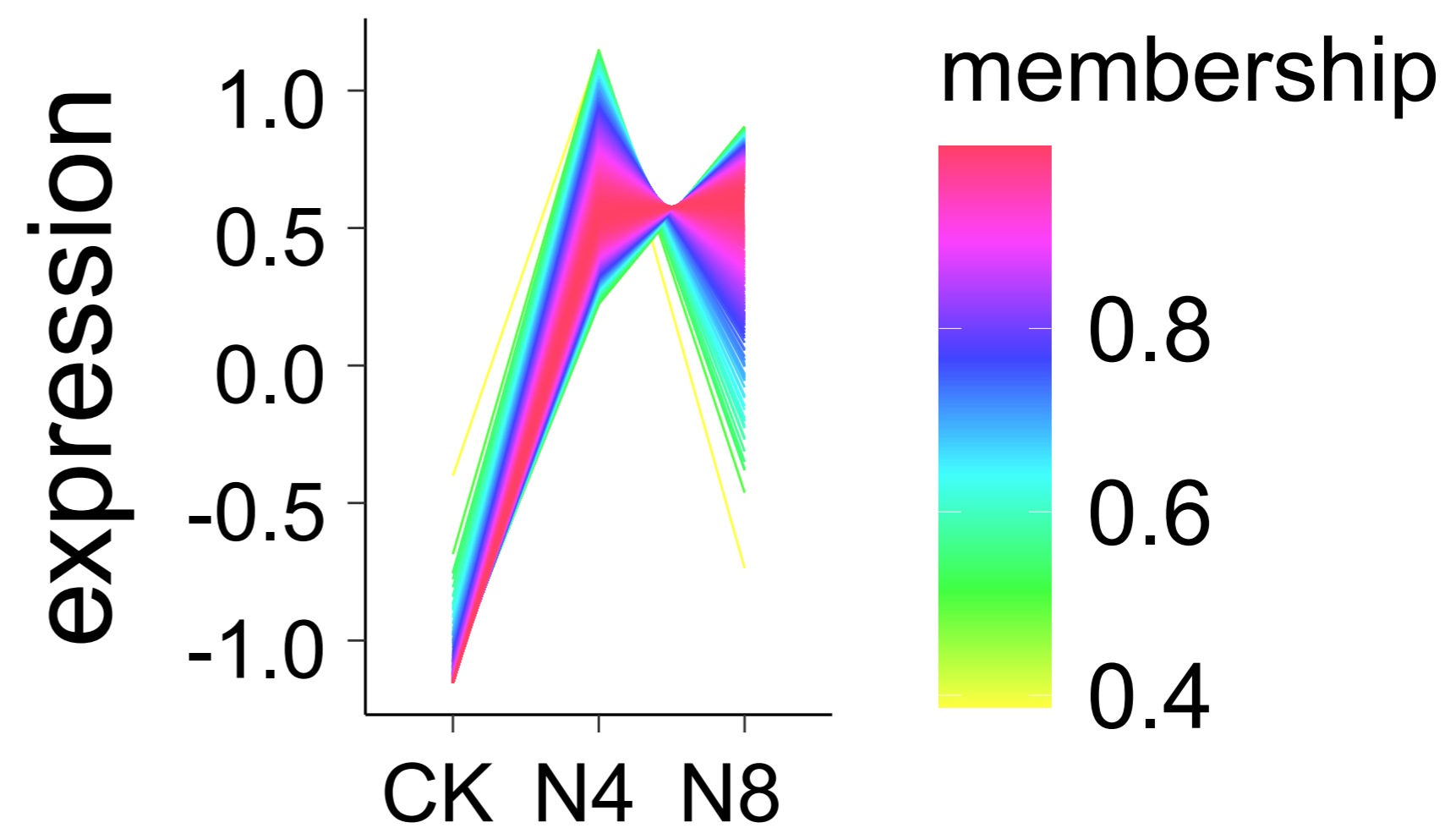

### Cluster 2

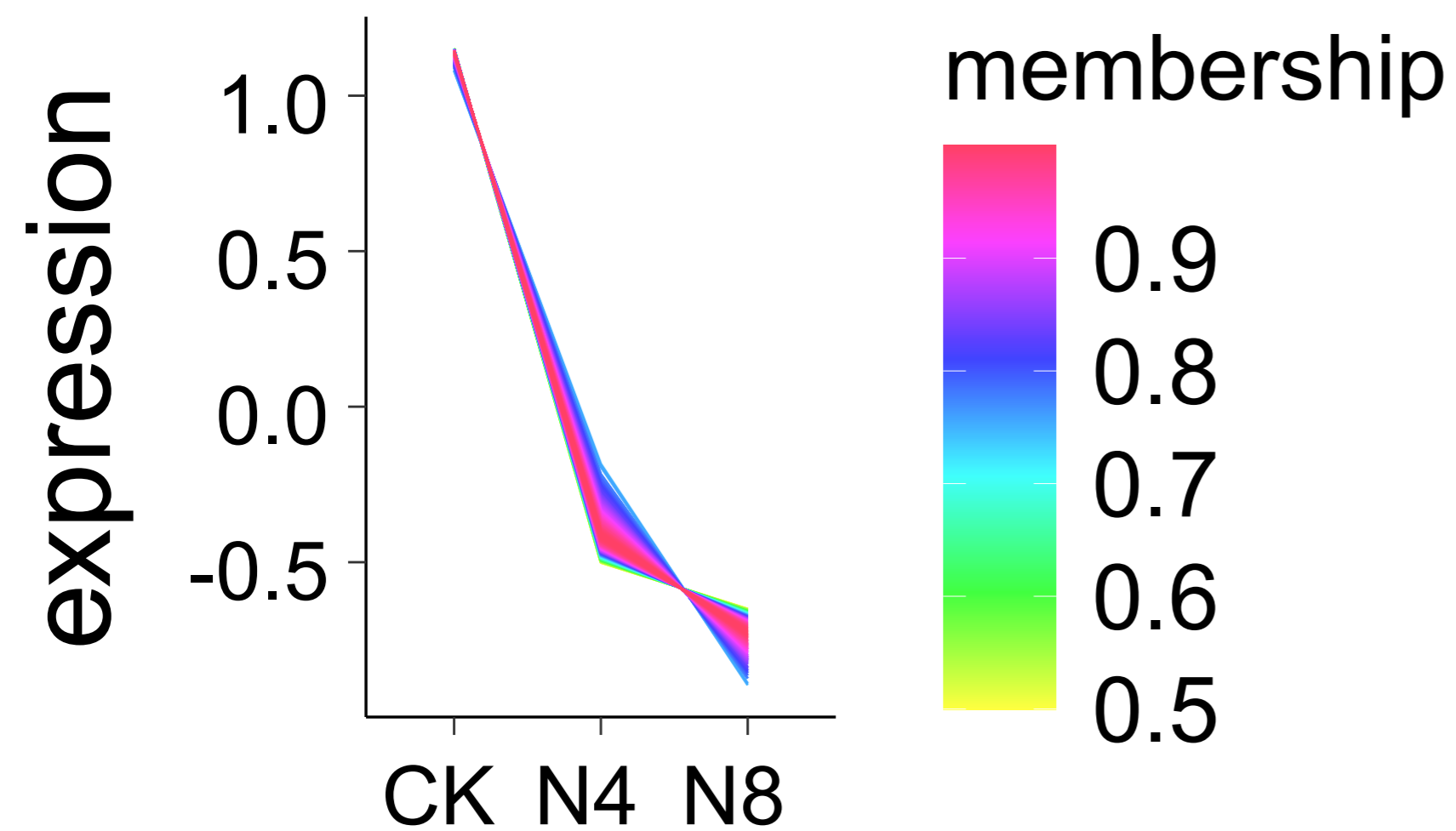

### Cluster 4

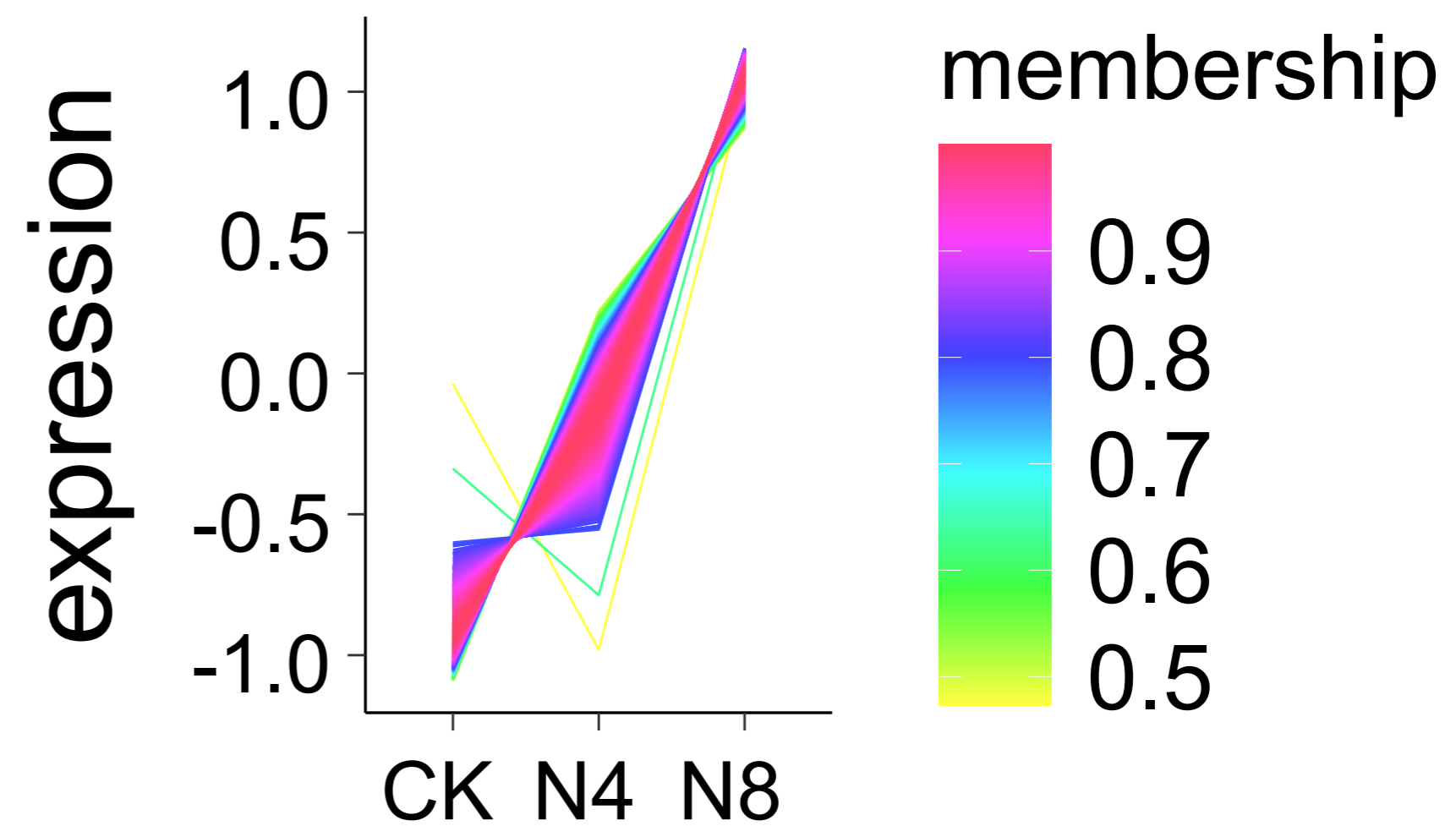

Supplement: Supplementary file 1 [file genes-17-00019-s001.zip › genes-4063062-supplementary/Supplementary File/Figure S3.pdf]

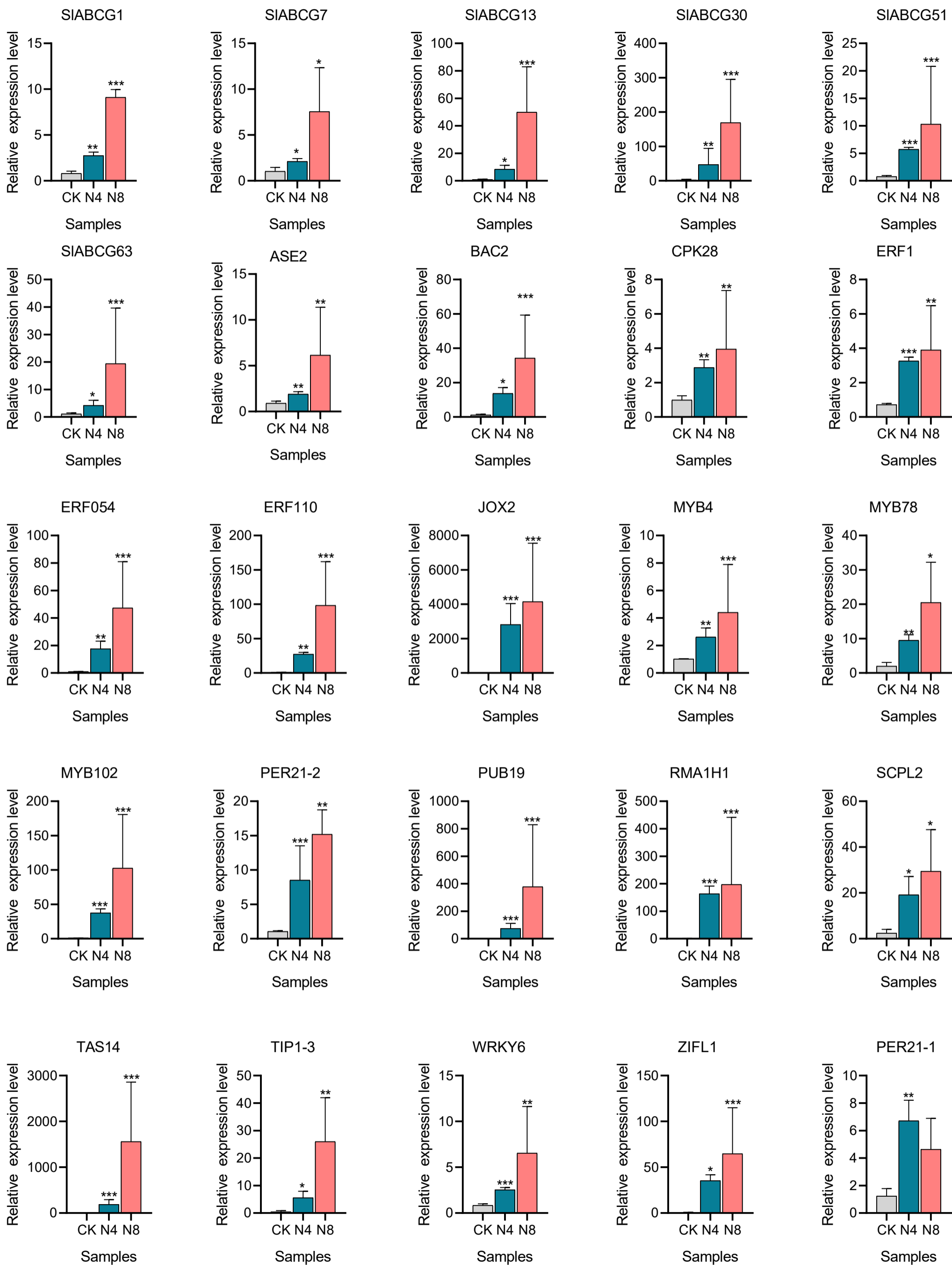

Supplement: Supplementary file 1 [file genes-17-00019-s001.zip › genes-4063062-supplementary/Supplementary File/Figure S4.pdf]
